# Statin Exposure and Survival Outcomes in Amyotrophic Lateral Sclerosis in an Asian Cohort

**DOI:** 10.1002/mus.70427

**Published:** 2026-10-12

**Authors:** Anushika Raheja, Kexin Ang, Bernett Lee, Seyed Ehsan Saffari, Eng King Tan, Crystal Jing Jing Yeo

**Affiliations:** ^1^ Department of Neurology National Neuroscience Institute (Singapore General Hospital Campus) Singapore Singapore; ^2^ Department of Neurology National Neuroscience Institute (Tan Tock Seng Campus) Singapore Singapore; ^3^ Singapore Immunology Network (SIgN) Agency for Science, Technology and Research (A*STAR) Singapore Singapore; ^4^ Centre for Biomedical Informatics, Lee Kong Chian School of Medicine Nanyang Technological University Singapore Singapore; ^5^ A*STAR Infectious Diseases Labs Agency for Science, Technology and Research A*STAR Singapore Singapore; ^6^ Institute of Molecular and Cell Biology (IMCB) Agency for Science, Technology and Research (A*STAR) Singapore Singapore; ^7^ School of Medicine, Medical Sciences and Nutrition University of Aberdeen Aberdeen UK

**Keywords:** amyotrophic lateral sclerosis, Asia, disease progression, statins, survival analysis

## Abstract

**Introduction:**

Data from Asian cohorts on whether statin therapy affects amyotrophic lateral sclerosis (ALS) progression are limited. We examined whether statin exposure was associated with ALS disease duration or tracheostomy‐free survival in a multiethnic Asian ALS cohort.

**Methods:**

Data from patients diagnosed with ALS between 2012 and 2025 were retrospectively reviewed. Patients were categorized as “ever” or “never” statin users for primary analyses, and as “never,” “former,” or “current” users at diagnosis for additional analyses. Disease duration was defined as time from symptom onset to death, and tracheostomy‐free survival as time from symptom onset to tracheostomy or death without tracheostomy. Kaplan–Meier and Cox regression analyses evaluated associations between statin exposure and both outcomes. Additional analyses examined statin discontinuation after diagnosis using time‐varying exposures. Sensitivity analyses excluded post‐diagnosis imitators and applied 30‐, 90‐, and 180‐day lags.

**Results:**

Among 103 ALS patients, 27 (26.2%) had ever received statins. Statin exposure was not significantly associated with disease duration (hazard ratio [HR]: 1.42, 95% CI: 0.64–3.14, *p* = 0.387) or tracheostomy‐free survival (HR: 1.04, 95% CI: 0.50–2.18, *p* = 0.913). Post‐diagnosis statin discontinuation was associated with shorter survival in a time‐varying analysis (HR: 6.52, 95% CI: 1.48–28.74, *p* = 0.013), although this finding was hypothesis‐generating. Sensitivity analyses did not show statistically significant associations.

**Discussion:**

In this multiethnic Asian ALS cohort, statin exposure was not significantly associated with disease duration or tracheostomy‐free survival. These findings add to the evidence that statins do not need to be discontinued due to ALS diagnosis. Larger studies are needed to define this association.

**Abbreviations**
ALS: amyotrophic lateral sclerosis
ALSFRS‐R: ALS Functional Rating Scale—revised
CI: confidence interval
EMR: electronic medical records
HR: hazard ratio
SD: standard deviation

## 1 Introduction

Dyslipidemia has been associated with slower disease progression and improved survival in amyotrophic lateral sclerosis (ALS) [1, 2]. It is uncertain if lipid‐lowering therapies such as statins, which may be myotoxic [2, 3, 4], influence ALS disease progression [5, 6].

Experimental data have suggested neuroprotective and neurotoxic effects of cholesterol‐lowering therapy in ALS patients, but clinical evidence remains minimal and inconsistent [7, 8, 9]. Early associations reflected surveillance bias, or reverse causation, where prodromal ALS symptoms prompted medical evaluation and statin initiation [10, 11, 12]. A Mendelian randomization study linked 3‐hydroxy‐3‐methylglutaryl‐coenzyme A reductase inhibition with ALS susceptibility at the genetic level, but this potentially reflects disease biology rather than clinical effects of statin exposure [13]. Evidence evaluating statin use after ALS onset is more limited, but generally consistent. Cohort studies from Canada and Norway report no association between statin exposure and ALS progression or survival, even after adjusting for demographic and clinical factors [5, 8].

As data from Asian ALS populations remain limited, we aim to describe the demographic and clinical characteristics of multiethnic ALS patients in Singapore and evaluate the association between statin exposure and disease progression and survival. We additionally examined statin status at ALS diagnosis and the association between statin discontinuation after diagnosis and survival.

## 2 Methods

### 2.1 Data Collection

Data on patients diagnosed with ALS who were followed at the National Neuroscience Institute at Tan Tock Seng, a tertiary adult neurology specialist clinic in Singapore, between 2012 and 2025 were collected. Patients were eligible for inclusion if they fulfilled the Gold Coast [14] diagnostic criteria for ALS. Patients without accessible electronic medical records (EMRs) or for whom research access was restricted were excluded from the study. Written informed consent was obtained for access to the patients' EMR. The study was approved by the Central Institution Review Board (CIRB 2018–2341).

Demographic data included age, sex, race, and date of symptom onset. Additional clinical data and statin exposure were retrieved from the EMR. Patients were categorized according to statin exposure as “never” or “ever” users, with “ever” users further classified as “former” users (statin discontinued before ALS diagnosis) or “current” users (statin use at ALS diagnosis). Baseline demographic and clinical characteristics were compared between exposure groups. Missing data were infrequent (< 5%) and handled using complete case analysis.

The primary outcomes were survival measures: (1) disease duration (time from symptom onset until death) and (2) tracheostomy‐free survival (time from symptom onset to either tracheostomy or death for those without tracheostomy). Patients who remained alive without any event occurrence were censored at the date of last available review of EMR (October 15, 2025). Secondary outcomes included functional decline, assessed using the Amyotrophic Lateral Sclerosis Functional Rating Scale—Revised (ALSFRS‐R) [15]. Slopes were calculated at baseline (48 − ALSFRS‐R score divided by time from symptom onset to diagnosis) and across the disease course (change in ALSFRS‐R score from first to last visit divided by follow‐up duration), expressed as monthly changes. Patients were classified as slow (> −0.5), intermediate (−0.5 to −1.5), or fast (< −1.5) progressors, based on overall ALSFRS‐R decline, with higher negative slopes indicating more rapid disease progression [16].

### 2.2 Statistical Analysis

Statistical analyses were performed using Excel (Microsoft, Redmond, WA, USA) and R software (Core Team 2024). Continuous variables were summarized as means, while categorical variables were expressed as counts (percentages). Variables were compared using Wilcoxon rank‐sum or Fisher's exact tests as appropriate. A *p* < 0.05 was considered statistically significant.

Kaplan–Meier survival curves and log rank tests were used to compare disease duration and tracheostomy‐free survival according to statin exposure. Cox proportional hazards models estimated hazard ratios (HRs) and 95% confidence intervals (CIs) for the association between statin exposure and disease duration and tracheostomy‐free survival. Crude and adjusted Cox models were performed, with adjusted models including age at onset, sex, site of onset, time to diagnosis, and baseline ALSFRS‐R slope. Firth‐penalized Cox regression was additionally done to account for sparse events in the statin‐exposed group. Statin exposure was analyzed as “ever” versus “never” use. For additional statin status analysis, Cox models compared “current” versus “never” users at the time of ALS diagnosis. Among “current” users, discontinuation after diagnosis was analyzed as a time‐varying covariate using counting‐process format to avoid immortal‐time bias. Sensitivity analyses included exclusion of post‐diagnosis statin initiators and lagged exposure definitions of 30, 90, and 180 days to address potential bias.

### 2.3 STROBE Reporting Guidelines

We used the STROBE reporting checklist when editing, included in Supplementary Materials [17].

## 3 Results

Of 105 ALS patients identified in the medical records, 103 met the inclusion criteria and were included in our analysis (two patients were excluded due to their EMRs containing highly restricted information which was inaccessible for research use). Of these, 27 had ever received statins. Atorvastatin was the most prescribed statin, followed by simvastatin, rosuvastatin, and pitavastatin (Supplementary Table 1).

Baseline demographic and clinical characteristics comparing “never” and “ever” statin users are presented in Table 1. Statin “ever” users were older at symptom onset compared to “never” users. No significant differences were observed in sex, ethnicity, site of onset, time to diagnosis, baseline ALSFRS‐R decline, overall disease progression, or progression category. Although disease duration and tracheostomy‐free survival were longer in the “never” than “ever” users, survival analyses did not suggest a significant association between statin exposure and either disease duration or tracheostomy‐free survival.

**TABLE 1:** Demographic and clinical characteristics of patients with ALS according to statin use.

Characteristic	“Never” (*n* = 76)	“Ever” (*n* = 27)	*p*
Age at symptom onset (years)	55.47 (17.19)	59.93 (9.55)	0.0242*
Time to diagnosis (months)	16.10 (24.45)	15.28 (17.32)	0.747
Disease duration (years)	6.16 (3.47)	4.00 (2.49)	0.0019*
Tracheostomy‐free survival (months)	66.35 (43.22)	45.55 (30.16)	0.0235*
ALSFRS‐R slope at baseline (/month)	−2.01 (2.55)	−1.01 (1.38)	0.0701
ALSFRS‐R slope over monitoring (/month)	−0.91 (0.84)	−0.72 (0.59)	0.381
Sex			0.104
Female	31 (40.8%)	6 (22.2%)	
Male	45 (59.2%)	21 (77.8%)	
Race			1
Caucasian	2 (2.6%)	0 (0.0%)	
Chinese	58 (76.3%)	21 (77.8%)	
Eurasian	1 (1.3%)	0 (0.0%)	
Indian	10 (13.2%)	4 (14.8%)	
Malay	5 (6.6%)	2 (7.4%)	
Site of onset			0.225
Limb	61 (81.3%)	25 (92.6%)	
Bulbar	14 (18.7%)	2 (7.4%)	
Progression category (*n* = 78)			0.747
Fast	9 (16.4%)	2 (8.7%)	
Intermediate	26 (47.3%)	12 (52.2%)	
Slow	20 (36.4%)	9 (39.1%)	

*Note:* Continuous variables are presented as mean (standard deviation) and categorical variables as *n* (%).

* *p* < 0.05.

### 3.1 Survival Analyses According to “Never” and “Ever” Statin Exposure

Kaplan–Meier analysis showed no significant difference in disease duration (Figure 1A) and tracheostomy‐free survival (Figure 1B) between “ever” and “never” statin users.

**FIGURE 1:**
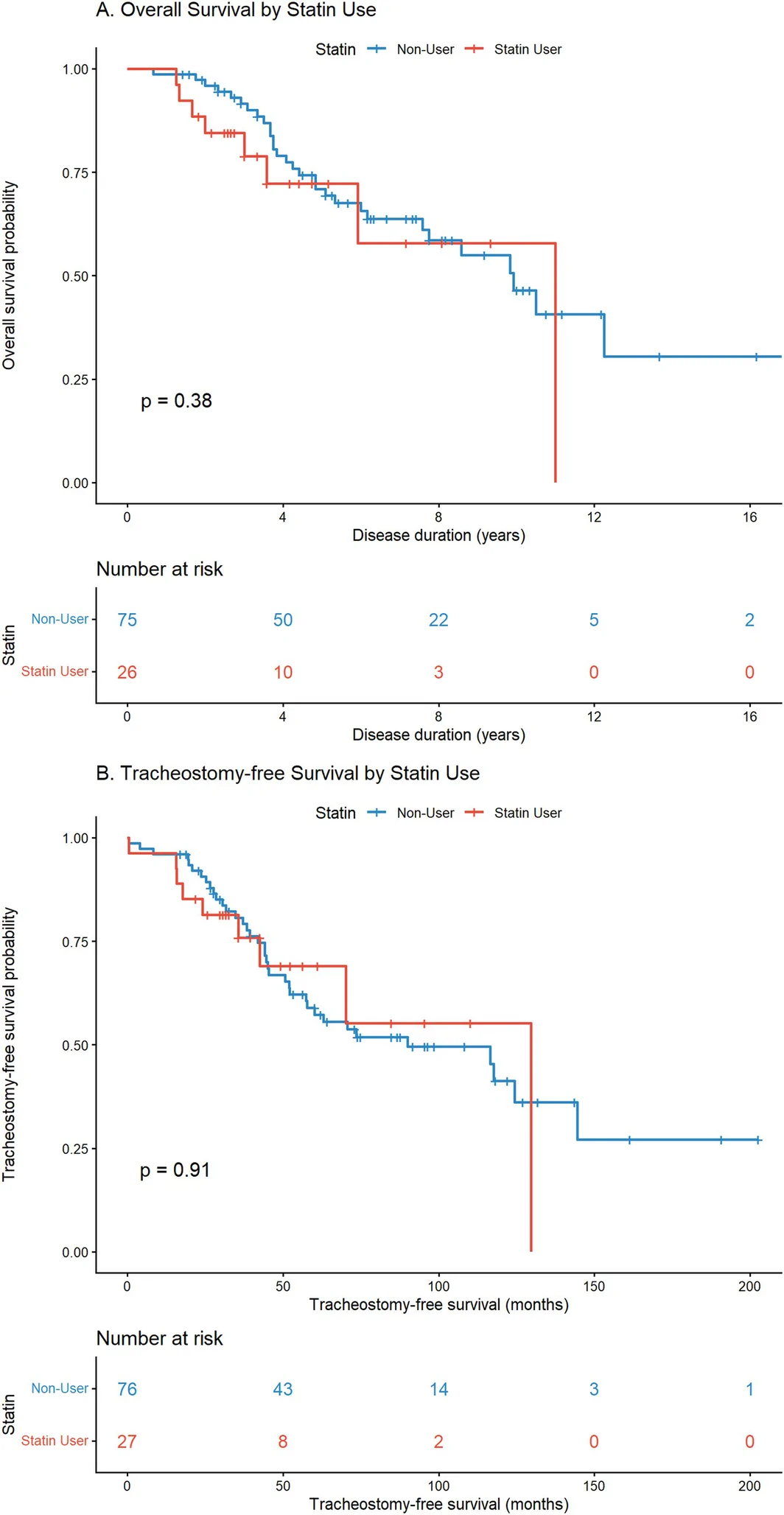
Kaplan–Meier survival curves for (A) disease duration and (B) tracheostomy‐free survival according to statin use. Log rank *p* values are shown for each panel.

Cox regression analyses are presented in Tables 2 and 3. Age at onset, site of onset, time to diagnosis and baseline ALSFRS‐R slope were significantly associated with tracheostomy‐free survival on univariable Cox analysis (Table 2). “Never” or “ever” statin exposure was not significantly associated with disease duration or tracheostomy‐free survival. Crude, Firth‐penalized Cox regression and adjusted Cox models for age at onset, sex, site of onset, and time to diagnosis confirmed no significant association between statin exposure and disease duration or tracheostomy‐free survival (Table 3).

**TABLE 2:** Univariable Cox proportional hazards analyses of clinical variables.

Variable	Disease duration HR (95% CI)	*p*	Tracheostomy‐free survival HR (95% CI)	*p*
Statin use (“ever” vs. “never”)	1.42 (0.64–3.14)	0.3870	1.04 (0.50–2.18)	0.9130
Age at onset	1.01 (0.99–1.03)	0.2850	1.01 (1.00–1.03)	0.0485*
Sex	0.57 (0.29–1.14)	0.1120	0.54 (0.29–1.02)	0.0564
Site of onset	1.23 (0.51–2.96)	0.6400	2.05 (1.01–4.15)	0.0457*
Time to diagnosis	0.98 (0.96–1.00)	0.0735	0.98 (0.96–1.00)	0.0348*
Baseline ALSFRS‐R slope	0.73 (0.51–1.06)	0.0979	0.50 (0.36–0.70)	4 × 10^−5^ *

*Note:* HR > 1 indicates greater hazard (shorter survival).

Abbreviations: CI, confidence interval; HR, hazard ratio.

* *p* < 0.05.

**TABLE 3:** Crude, Firth penalized, and adjusted Cox models for statin use.

Endpoint	Crude HR (95% CI), *p*	Firth HR (95% CI), *p*	Adjusted HR (95% CI), *p*
Disease duration	1.42 (0.64–3.14), *p* = 0.387	1.48 (0.64–3.08), *p* = 0.336	1.59 (0.69–3.70), *p* = 0.278
Tracheostomy‐free survival	1.04 (0.50–2.18), *p* = 0.913	1.08 (0.50–2.14), *p* = 0.829	1.13 (0.52–2.45), *p* = 0.750

*Note:* Adjusted models include age at onset, sex, site of onset, and time to diagnosis.

Abbreviation: Firth, Firth penalized Cox regression (used to address sparse events in the statin‐exposed group).

Additional analysis by statin status at diagnosis showed no significant differences in disease duration or tracheostomy‐free survival between “current” and “never” users. The three “former” users had no deaths or tracheostomy‐free survival events and are reported descriptively only. Cox models therefore compare “current” versus “never” users. Kaplan–Meier analysis showed no significant difference in disease duration (Figure 2A) and tracheostomy‐free survival (Figure 2B) between “current” and “never” statin users.

**FIGURE 2:**
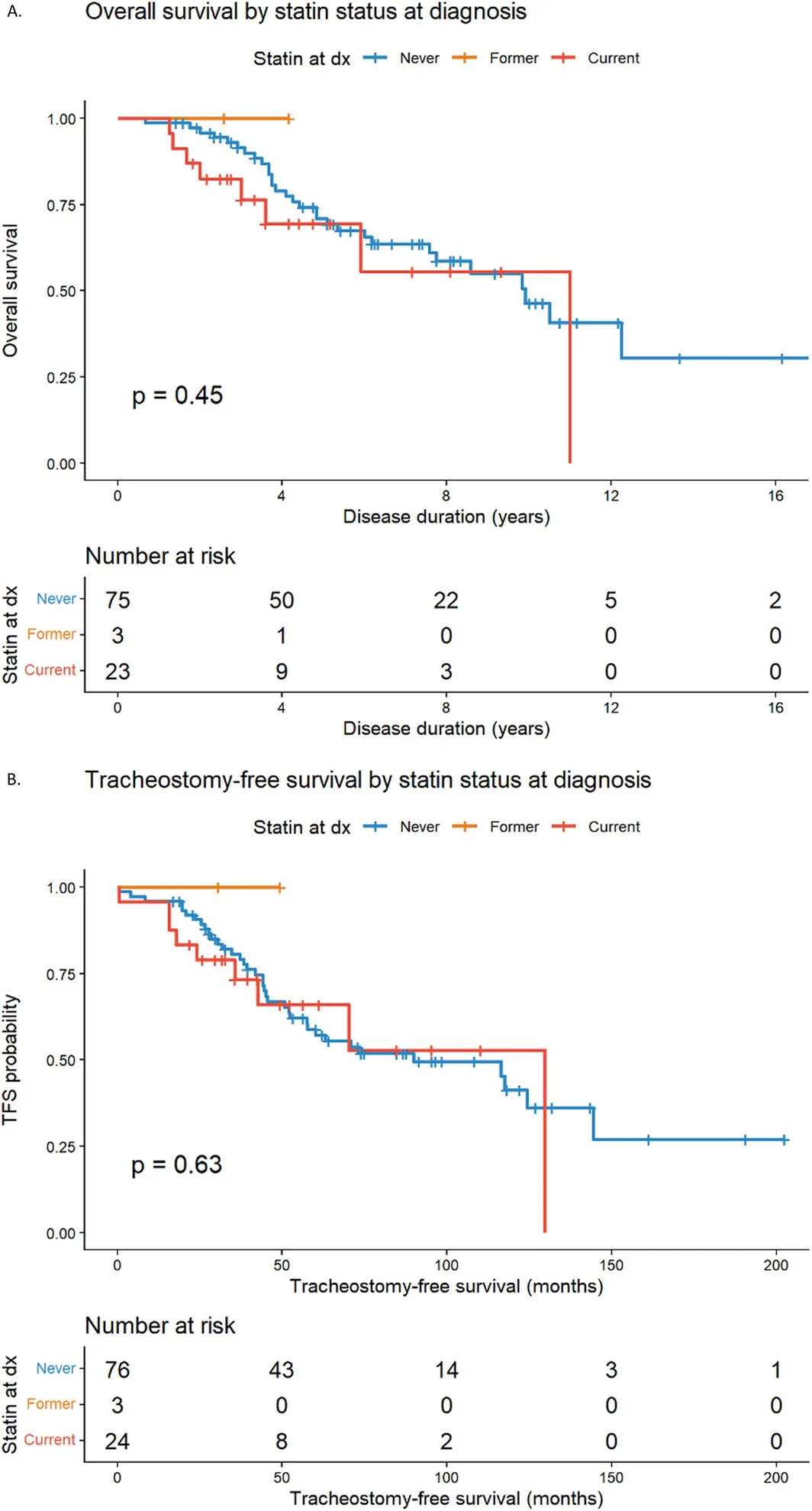
Kaplan–Meier survival curves for (A) disease duration and (B) tracheostomy‐free survival according to statin use at diagnosis. Log rank *p* values are shown for each panel.

Among “current” users, discontinuation occurs after diagnosis; it was therefore modeled as a time‐varying covariate (counting‐process format) to avoid immortal‐time bias. The analysis was based on seven discontinuers and nine events. Post‐diagnosis statin discontinuation was associated with shorter survival (Table 4).

**TABLE 4:** Statin status at diagnosis and survival outcomes.

Statin status at diagnosis	*N*	Deaths	TFS events
Never	76	31	37
Former	3	0	0
Current	24	9	9

Endpoint	Comparison	HR (95% CI)	*p*
Disease duration	Current vs. never	1.52 (0.69–3.36)	0.296
Tracheostomy‐free survival	Current vs. never	1.14 (0.55–2.38)	0.728
Survival, time‐varying	Off‐ vs. on‐statin	6.52 (1.48–28.74)	0.013*

*Note:* Former users are reported descriptively due to no deaths or tracheostomy events in this group. Cox models comparing current versus never users excluded former users.

* *p* < 0.05.

Sensitivity analyses excluding patients who initiated statins after ALS diagnosis and applying lagged exposure definitions of 30, 90, and 180 days did not show statistically significant associations between statin exposure and disease duration and tracheostomy‐free survival (Table 5).

**TABLE 5:** Sensitivity analyses of statin use and survival outcomes.

Scenario	Endpoint	HR (95% CI)	*p*
Exclude post‐diagnosis initiators	Disease duration	3.11 (0.68–14.22)	0.1430
	Tracheostomy‐free survival	1.49 (0.34–6.45)	0.5930
30‐Day lag	Disease duration	3.68 (0.81–16.75)	0.0925
	Tracheostomy‐free survival	1.80 (0.42–7.79)	0.4320
90‐Day lag	Disease duration	3.68 (0.81–16.75)	0.0925
	Tracheostomy‐free survival	1.80 (0.42–7.79)	0.4320
180‐Day lag	Disease duration	3.68 (0.81–16.75)	0.0925
	Tracheostomy‐free survival	1.80 (0.42–7.79)	0.4320

*Note:* 30‐, 90‐, and 180‐day lag analyses gave identical results, as no statin user started therapy within 180 days of their event.

## 4 Discussion

In our multiethnic Asian cohort of 103 ALS patients, Kaplan–Meier and Cox analyses showed no significant association between statin exposure and either disease duration or tracheostomy‐free survival. The lack of a statistically significant association was consistent across crude, Firth‐penalized, and adjusted Cox models. Statin status at diagnosis also shows “current” users to not have significantly different disease duration or tracheostomy‐free survival compared with never users, providing additional evidence that statin use at the time of ALS diagnosis was not associated with worse survival outcomes in this cohort.

Post‐diagnosis statin discontinuation was associated with shorter survival. The analysis was based on seven discontinuers and nine events. Although statistically feasible, the estimate is imprecise and highly susceptible to confounding by indication, because statins in ALS are frequently withdrawn during clinical decline or transition to end‐of‐life care [5, 18]. Discontinuation may therefore be regarded as a marker of clinical deterioration rather than a cause of shortened survival, and this result is presented as hypothesis‐generating only.

Initial concerns linking statins to ALS were based on early case reports and observations/surveillance suggesting possible neurotoxicity or increased ALS risk [19, 20]. However, subsequent pharmacoepidemiologic analyses have not supported these claims [21]. A Danish national registry study found that pre‐diagnosis statin use did not increase risk of ALS onset or worsen clinical progression [10]. More recent data from China showed mixed results, with no overall harmful effect but a potential association between high‐intensity statins and slightly faster functional decline [13]. Altogether, these studies suggest that statins are unlikely to have meaningful negative effects on ALS progression, and our findings on a Southeast Asian population can be interpreted alongside this broader body of evidence.

A Norwegian study also examined statin exposure at ALS diagnosis and found no association between statin use and ALS survival. Patients who discontinued statins in the year before diagnosis had poorer prognosis than those who continued therapy, while avoiding statin use after diagnosis was unassociated with survival [5]. These findings are consistent with our analysis showing no significant difference in survival between “current” and “never” statin users at diagnosis. This highlights that statin discontinuation may reflect underlying disease progression rather than a causal effect of treatment.

Statin therapy varied in type and dose among patients, and some initiated statins after ALS diagnosis, making direct comparison with studies evaluating long‐term or high‐intensity exposure difficult. We additionally performed sensitivity analyses excluding post‐diagnosis statin initiators and applying lagged exposure definitions of 30, 90, and 180 days. These analyses did not show significant association between statin exposure and either disease duration or tracheostomy‐free survival, although the wide confidence intervals limit the estimates' precision.

This study provides regional data on statin effects in ALS from Southeast Asia, where ALS epidemiology, lipid profiles, and cardiovascular risk factors differ from Western cohorts [22, 23]. However, limitations should be acknowledged. Our modest sample size, particularly the small number of statin users, limited statistical power and resulted in imprecise effect estimates with wide confidence intervals. Although adjusted analyses were performed, residual and unmeasured confounding from factors including cardiovascular disease severity, premorbid cholesterol levels, or lifestyle factors cannot be excluded. The time‐varying discontinuation analysis was limited by the small number of patients who discontinued statins after diagnosis, making the estimate susceptible to random error and confounding by indication. Duration of statin use varied among patients. Finally, our patients were recruited from a single neurology clinic, and therefore our findings may not be generalized to the broader Southeast Asian ALS population.

Despite these limitations, our study adds to larger epidemiological studies, which have generally reported no adverse effect of statins on ALS incidence, functional decline, or survival [24]. Our findings do not provide evidence that statins should be discontinued when patients are diagnosed with ALS, particularly when used for primary or secondary cardiovascular protection. The association between post‐diagnosis statin discontinuation and shorter survival is hypothesis‐generating and should not be interpreted as a causal effect of discontinuation, given the small number of discontinuers and the potential for discontinuation to reflect clinical deterioration. Future multicenter Asian studies with larger sample sizes and detailed assessment of cumulative statin exposure are needed to better define this relationship.

## 5 Conclusion

In this multiethnic Singaporean ALS cohort, no significant association was observed between statin exposure and either disease duration or tracheostomy‐free survival. Our findings add to existing evidence that statin use does not influence ALS progression, and there is no evidence from our study that statins should be discontinued when indicated for primary or secondary cardiovascular protection. Larger multicenter Asian studies are needed to clarify this association.

## Author Contributions

**Anushika Raheja:** investigation, writing – original draft, methodology, writing – review and editing. **Kexin Ang:** writing – review and editing. **Bernett Lee:** supervision, formal analysis, writing – review and editing. **Seyed Ehsan Saffari:** writing – review and editing, formal analysis, resources, data curation. **Eng King Tan:** writing – review and editing. **Crystal Jing Jing Yeo:** conceptualization, funding acquisition, writing – review and editing, resources, project administration.

## Funding

This work was supported by Agency for Science, Technology and Research (A*STAR) (C210112024) and National Neuroscience Institute Singapore (IRNMR21CPGJJ).

## Ethics Statement

We confirm that we have read the Journal's position on issues involved in ethical publication and affirm that this report is consistent with those guidelines.

## Conflicts of Interest

The authors declare no conflicts of interest.

## Supporting information


**Supplementary Table 1** Doses and types of statins used in our cohort.

**Data S1:** Supporting Information.

## Data Availability

The data that support the findings of this study are available on request from the corresponding author. The data are not publicly available due to privacy or ethical restrictions.
